# Baltimore Quartet

**Published:** 1875-04

**Authors:** 


The Baltimore Quartet.—For once, Baltimore has a genuine sensation, and the medical profession, as well as upper and lower Ten-dom, is making the most of it. On Tuesday, February 16, Dr. Dausch, assisted by Dr. Mansfield, delivered Mrs. Hahn, 119 Low Street, of *four* daughters. The first child presented by the right shoulder, the other three were footlings. There were two placentæ, three children being attached to one, and one having its own independent supply. The large placenta was about fifteen inches in diameter, and on the fœtal surface had the appearance of three ordinary placentæ, with a cord in the centre of each, placed so as to form a circle, thus $\overset{\text{O}}{\text{OO}}$, but the maternal surface showed no marks of division, and they could not be separated without laceration. The labor occupied about an hour and a half, and was free from pain. Ergot was given in ʒij. doses, but produced no contractions of the uterus.

The mother is small, and in an advanced stage of consumption. This was her third confinement, and both her other children were breech-presentations, and the labors free from pain. The quartet weigh about twenty-four pounds, and the placentæ about four pounds, which gives about thirty pounds as the weight of the uterine contents before delivery. The children are strong and healthy, and the father is doing a good business exhibiting them to the curious, for which he charges an admission fee. Collections have been made at the hotels and other places for the benefit of this interesting family. It is intimated that a cow would be a very acceptable present.—*Medical Times, March* 20.

An Affecting Dissecting-Room Scene.—The janitor of an Indianapolis medical college was deeply affected on recognizing his mother-in-law on the dissecting-table. His grief was the more poignant from the fact that he had himself carried the stolen corpse up three flights of stairs.—*Medical Record.*

